# Enhancing Safety in Regional Anesthesia: Guidelines from the Italian Society of Anesthesia, Analgesia, Resuscitation and Intensive Care (SIAARTI)

**DOI:** 10.1186/s44158-025-00245-y

**Published:** 2025-05-14

**Authors:** Vito Torrano, Salvatore Anastasi, Eleonora Balzani, Enrico Barbara, Astrid Ursula Behr, Mario Bosco, Claudio Buttarelli, Silvia Bruletti, Dario Bugada, Chiara Cadeddu, Gianluca Cappelleri, Luigi Cardia, Salvatore Casarano, Andrea Cortegiani, Floriana D’Ambrosio, Miryam Del Vicario, Andrea Fanelli, Pierfrancesco Fusco, Giuseppe Gazzerro, Daniela Ghisi, Antonino Giarratano, Fabio Gori, Massimiliano Greco, Paolo Angelo Grossi, Alberto Manassero, Gianluca Russo, Salvatore Sardo, Cosimo Savoia, Marco Tescione, Giulia Tinti, Alessandro De Cassai

**Affiliations:** 1 Department of Anesthesia, Critical Care and Pain Medicine, ASST Grande Ospedale Metropolitano Niguarda, Milan, Italy; 2 Department of Anesthesiology and Intensive Care, Nesima, Catania Italy; 3 Department of Surgical Sciences, University of Turin, Turin, Italy; 4 Department of Anesthesiology and Intensive Care, Humanitas Mater Domini, Castellanza, Varese, Italy; 5 Department of Anesthesiology and Intensive Care, ULSS 6 Euganea, Padua, Italy; 6 Department of Anesthesiology and Intensive Care, ASL Roma 1, Rome, Italy; 7 Institute of Anesthesia, Treviso Hospital, Treviso, Italy; 8 Cardinal Gusmini Foundation-ONLUS, Vertova, Bergamo, Italy; 9 Department of Emergency and Critical Care, ASST Papa Giovanni XXIII, Bergamo, Italy; 10 Erasmus School of Health Policy and Management, Erasmus University Rotterdam, Rotterdam, Netherlands; 11 Department of Anesthesia, Intensive Care and Pain Medicine, Monza Polyclinic, Monza, Monza-Brianza, Italy; 12 Department of Pain Medicine, University Hospital “Gaetano Martino”, Messina, Italy; 13 Department of Human Pathology “G. Barresi”, University of Messina, Messina, Italy; 14 Anesthesiology Department, Pia Fondazione Tricase, Lecce, Italy; 15 Department of Anesthesia Intensive Care and Emergency, Policlinico Paolo Giaccone, Palermo, Italy; 16 Department of Precision Medicine in Medical, Surgical and Critical Care Area (Me.Pre.C.C.), University of Palermo, Palermo, Italy; 17 Section of Hygiene, University Department of Life Sciences and Public Health, Università Cattolica del Sacro Cuore, Rome, Italy; 18 Department of Anesthesiology and Intensive Care Medicine, “Agostino Gemelli” University Polyclinic Foundation IRCCS — Catholic University of The Sacred Heart, Rome, Italy; 19 Department of Anesthesia, Intensive Care and Pain Medicine, SS. Filippo E Nicola Hospital, Avezzano, L’Aquila Italy; 20 Department of Anesthesiology, Intensive Care and Pain Medicine, AORN Dei COLLI Monaldi-CTO, Naples, Italy; 21 Department of Anesthesiology and Intensive Care 1, Perugia Hospital, Perugia, Italy; 22 Department of Biomedical Sciences, Humanitas University, Milan, Italy; 23 Department of Anesthesiology and Intensive Care, IRCCS Humanitas Research Hospital, Milan, Italy; 24 Anesthesia, Critical Care and Pain Medicine Consultant, ASST Gaetano Pini-CTO, Milan, Italy; 25 Unit of Anesthesia, Casa Di Cura Città Di Bra, Cuneo, Italy; 26 Anesthesia and Intensive Care, ASST Lodi, Lodi, Italy; 27 Department of Medical Sciences and Public Health, University of Cagliari, Cagliari, Italy; 28 Department of Anesthesiology and Critical Care, Bianchi-Melacrino-Morelli Health Institute of Reggio Calabria, Reggio Calabria, Italy; 29 School of Medicine and Surgery, University of Milano-Bicocca, Milan, Italy; 30 Department of Medicine (DIMED), University of Padua, Padua, Italy; 31 Institute of Anesthesia and Intensive Care, University Hospital of Padua, Padua, Italy

**Keywords:** Regional anesthesia, Patient safety, Anticoagulation, Neuraxial blocks, Ultrasound guidance

## Abstract

**Background:**

Regional anesthesia techniques have become integral to modern perioperative care, offering enhanced pain management and recovery outcomes. However, their application in patients with specific conditions, such as anticoagulation therapy or preexisting comorbidities, raises concerns regarding safety and efficacy. Current guidelines addressing these issues are fragmented, necessitating comprehensive, evidence-based recommendations.

**Methods:**

A multidisciplinary panel of experts in anesthesiology and pain management was convened under the auspices of the Italian Society of Anesthesia, Analgesia, Resuscitation, and Intensive Care (SIAARTI). The guidelines presented herein were developed according to the GRADE system (Grading of Recommendations of Assessment Development and Evaluations), in compliance with the methodological manual for the production of clinical practice guidelines published by the National Center for Clinical Excellence, Quality, and Safety of Care, Italian National Institute of Health.

**Results:**

The guidelines encompass recommendations on neuraxial blocks in anticoagulated patients, the dual guidance use in peripheral nerve blocks, the role of sterile field preparation, and post-procedural monitoring. Evidence from meta-analyses and large-scale observational studies supported most recommendations, though limitations in study heterogeneity were noted.

**Conclusions:**

These guidelines provide a structured framework for clinicians to enhance patient safety and procedural efficacy in regional anesthesia. Further research is encouraged to address identified gaps, particularly regarding specific patient subgroups and novel regional anesthesia techniques.

**Supplementary Information:**

The online version contains supplementary material available at 10.1186/s44158-025-00245-y.

## Introduction

Regional anesthesia (RA) is a cornerstone of modern anesthetic practice, offering significant advantages in perioperative care through improved pain control, reduced opioid consumption, and enhanced recovery [1]. The introduction of ultrasound guidance has significantly advanced the practice of RA, improving the precision, safety, and efficacy of these techniques.

The growing adoption of RA highlights the need for robust educational frameworks that ensure both theoretical understanding and practical competency [2]. Despite its benefits, the adoption of RA is often met with concerns regarding safety in special populations, such as patients on anticoagulant therapy or those undergoing high-risk procedures [3]. Additionally, the rapid proliferation of newly described RA techniques in recent years reflects growing interest from researchers but often lacks comprehensive exploration of their mechanisms of action [4]. To ensure consistent safety and efficacy across varied clinical contexts, it is imperative to develop and adhere to clear, evidence-based guidelines.

In this context, the Italian Society of Anesthesia, Analgesia, Resuscitation, and Intensive Care (SIAARTI) convened a multidisciplinary panel of experts to address the critical challenges and unanswered questions in RA practice. These guidelines aim to provide clinicians with actionable recommendations for the safe and effective use of RA, covering topics such as neuraxial and peripheral nerve blocks, the integration of ultrasound and neurostimulation techniques, and infection prevention strategies. Specific focus is placed on mitigating risks associated with anticoagulation therapy, optimizing postoperative monitoring, and addressing potential complications like local anesthetic systemic toxicity (LAST).

This research did not receive any specific grant from public, commercial, or nonprofit organizations.

## Methods

The intended users of this guideline are specialists in anesthesia and resuscitation, while the target population includes adult patients (≥ 18 years) undergoing surgical procedures with regional anesthesia (RA) or benefiting from RA techniques. It is important to note that this document is the English translation of the original guidelines published in Italian. The complete version in the original language is accessible on the “Italian National Institute of Health” platform [5] and the SIAARTI website [6].

## Panel composition

The working group is composed of individuals with expertise in anesthesia and resuscitation, emergency and urgent care, nursing, legal matters, and public health.

Experts appointed by SIAARTI were selected based on their proven clinical, professional, and/or scientific experience. Other professionals were involved through national scientific societies accredited by the Ministry of Health, in accordance with Law No. 24 of March 8, 2017 [7], on safety of care and assisted persons, as well as professional liability for healthcare practitioners.

Specifically, on April 17, 2021, SIAARTI formally requested the participation of two experts from the presidents of the following scientific societies to contribute to the drafting process: *AICO (Italian Association of Operating Room Nurse)* and *ESRA-Italy (European Society of Regional Anesthesia-Italian Section)*. All invited societies joined the project, appointing delegates with proven clinical, professional, and/or scientific expertise.

Additionally, SIAARTI identified other technical-scientific figures to support the process and the experts. Specifically a methodologist, responsible for overseeing and ensuring the methodological process of this document, was selected based on specific skills detailed in their curriculum vitae; experts involved in the Evidence Review Team where chosen through a public call by SIAARTI, during which qualifications and competencies were evaluated, these experts have proven experience as literature search specialists in evidence retrieval and evaluation; lastly external reviewers with proven expertise in the subject matter and in applying clinical methodology.

Finally, to ensure the proper execution of the entire guideline process, a *Technical-Scientific Committee* was established. It includes the panel coordinators, two panel experts (with extensive experience in systematic literature reviews and the topic of this guideline), the methodologist, and seven literature search specialists.

## Panel interactions and decision-making processes

During the panel’s initial plenary meeting, the experts were presented with the methodological framework as defined by the National Guideline System (SNLG).

On May 12, 2021, the experts participated in a *scoping workshop* (led by the first author V. T.) to define the main topics addressed in this guideline.

At the end of the meeting, the experts provided their individual evaluations of the priority of each proposed item using an anonymous online form. The evaluation was based on a scale from 1 to 9, with 9 indicating maximum priority and 1 indicating low importance. Items that achieved over 75% agreement within the interquartile range (IQR) of 7–9 were included in this guideline.

### Working process

The expert panel was later divided into multidisciplinary working subgroups, each assigned specific items. These subgroups collaborated with the coordinators to propose PICO questions following the GRADE methodology.

The fundamental questions were structured using the PICO model (population, intervention, comparators, outcome):

- Population: Adult patients undergoing noncardiac surgery
- Intervention: Perioperative hemodynamic optimization interventions
- Comparator: Standard therapeutic management
- Outcome: Key and significant outcomes, including those critical for assessing the overall quality of evidence and the balance between benefits and risks (e.g., mortality and morbidity).

All PICO proposals were presented to the panel in a plenary session, after which panelists cast blind votes through an online form. Opinions were expressed using a Likert ordinal scale, following the UCLA-RAND method, where as follows:

- Scores 1–3 indicated rejection/disagreement (“not appropriate”).
- Scores 4–6 indicated “uncertainty.”
- Scores 7–9 indicated “appropriateness.”

Two rounds of voting were conducted, and only PICO questions that reached 75% agreement in the IQR range of 7–9 (appropriate) were approved.

The results of the clinical question votes were shared with the panel, which then reviewed and approved the list of outcomes for each question during a plenary session.

### Systematic literature review

For each PICO, a systematic literature review was conducted. After an initial screening of titles, abstracts, and full texts of the literature, the absence of supporting evidence and/or association measures for certain clinical questions prevented the application of the GRADE method and, consequently, the formulation of recommendations.

In light of this gap, the expert panel deemed it essential to address the relevant questions collectively, even without supporting evidence.

After completing the literature review and grading process, the expert panel reviewed the GRADE results and convened to develop recommendations and rationales wherever possible. For questions where evidence synthesis and grading were not feasible, the UCLA-RAND method was employed. GRADE evaluation is available as Table 1.

**Table 1:** GRADE outcomes

Quality assessment							Result summary				
**Study number**	**Study design**	**Risk of bias**	**Inconsistency**	**Indirectness**	**Imprecision**	**Other considerations**	**Patient number**				
							**Intervention**	**Comparison**	**Relative (95% *CI*)**	**Absolute (95% *CI*)**	**Effect**
Outcome 1: Incidence of spinal hematomas											
4	Observational studies	Not important	Not important	Serious	Not important		0/644 (0.0%)	0/2241 (0.0%)	Not estimable		Critical
Outcome 2: Reduction of the incidence of severe neurological and nervous complications											
11	RCTs	Serious	Not important	Not important	Not important		0/545 (0.0%)	0/546 (0.0%)	Not estimable		Critical
2	Observational studies	Not important	Serious	Not important	Not important		1/9238 (0.0%)	13/5499 (0.2)%			Critical
Outcome 3: Incidence of LAST											
6	RCTs	Serious	Not important	Serious	Not important		12/240 (5.0%)	4/211 (1.9%)			Critical
10	Observational studies	Not important	Not important	Not important	Not important		12/44,735 (0.0%)	17/30,587 (0.1%)			Critical
Outcome 4: Incidence of major bleeding events											
1	Observational studies	Not important	Serious	Very serious	Not important		36 cases	33 controls	Not estimable		Critical
Outcome 5: Onset of infections											
3	RCTs	Serious	Serious	Serious							
1	Observational studies	Not important	Serious	Serious					Not estimable		Critical
									Not estimable		Critical
Outcome 6: Onset of infections											
1	RCTs	Not important	Not important	Very serious			9/58 (15.5%)	18/55 (32.7%)			Critical
1	Observational studies	Not important	Serious	Very serious			5 cases	110 controls			Critical

A summary table which reports all PICOs with the corresponding recommendation (Table 2) is provided for reference.

**Table 2:** Summary table

No	PICO	Recommendation	Strength of recommendation
1	Is it safe to perform a central neuraxial block in patients on cardioaspirin therapy?	In adult patients on cardioaspirin therapy undergoing RA with neuraxial block, it is recommended to continue cardioaspirin administration, as it does not increase the incidence of spinal hematomas	Low
2	Does the combined use of ultrasound and nerve stimulation (ENS) in peripheral nerve blocks with a motor component in adult patients increase the efficacy and safety of surgical blocks, reducing neurological complications and nerve damage?	In adult patients undergoing medical procedures requiring RA, the use of dual guidance compared to ENS-only guidance in peripheral nerve blocks with a motor component is recommended, as it reduces the risk of serious neurological complications and/or nerve damage	Moderate
3	Is the use of ultrasound-guided peripheral block techniques safe in adult patients taking direct oral anticoagulants (DOAC)?	In adult patients taking DOACs, it is suggested to use ultrasound-guided peripheral nerve blocks, as it is safe and does not increase hemorrhagic complications	Low
4	Can the use of ultrasound-guided techniques increase safety in nerve block procedures in adult patients?	In adult patients, the use of ultrasound-guided techniques for the performance of peripheral nerve blocks is suggested, as it ensures the safety of the procedure and reduces the risk of complications compared to nerve blocks without ultrasound guidance	Moderate
5	Can a sterile surgical field (using disinfectants, covers, gloves, and sterile drapes) set up for the execution of neuraxial techniques, help reduce the occurrence of infections related to the technique itself?	In adult patients undergoing epidural anesthesia, the preparation of a sterile surgical field (using antiseptic, covers, gloves, drapes, and mask) is suggested to ensure patient safety	Low
6	Can a sterile surgical field setup (using disinfectants, covers, gloves, and sterile drapes) for the execution of continuous peripheral regional anesthesia techniques help reduce the development of infections related to the procedure itself?	In adult patients undergoing continuous peripheral regional anesthesia techniques, the preparation of a sterile surgical field (using disinfectant, covers, gloves, drapes, and mask) for the procedure is suggested to reduce the occurrence of infections and ensure patient and procedural safety	Low
**No**	**PICO**	**Good practice statement**	
7	Is the use of antiplatelet drugs safe for patients undergoing a dual guidance peripheral nerve block?	7.1 Superficial peripheral blocks are considered safe in patients on antiplatelet therapy regardless of the dosage or the drug taken 7.2 For deep peripheral nerve blocks in patients undergoing antiplatelet therapy, the same recommendations as neuraxial procedures should be followed regarding the suspension timing of antiplatelet drugs	
8	Is it safe to perform peripheral nerve block techniques with dual guidance in adult patients on anticoagulant therapy?	8.1 The expert panel suggests that whenever possible, an appropriate suspension period for anticoagulant medications is always preferable for both deep and superficial peripheral nerve blocks 8.2 The expert panel suggests that deep peripheral nerve blocks (such as lumbar plexus block) are high-risk procedures for bleeding, and, therefore, in the absence of an adequate suspension period from anticoagulant medications, these blocks cannot be performed safely 8.3 The expert panel suggests that superficial peripheral nerve blocks can be performed safely in adult patients receiving anticoagulants, even if these medications cannot be suspended 8.4 The expert panel suggests that the use of dual guidance, compared to not using it, should not influence the anticoagulant suspension time, as there is no evidence that the use of dual guidance is able to reduce hemorrhagic complications in anticoagulated patients 8.5 The expert panel suggests that all recommendations, precautions, and prescriptions outlined in international guidelines applied to nonurgent cases should be followed also in urgent and emergency situations	
9	Can the use of infusion pressure monitoring techniques, during the performance of peripheral blocks, reduce the onset of neurological complications in adult patients undergoing RA techniques?	9.1 The multidisciplinary expert panel believes that performing peripheral blocks with infusion pressure monitoring techniques does not reduce occurrence of neurological complications in adult patients undergoing RA techniques	
10	Can the preparation of a sterile surgical field (disinfectant, probe covers, gloves, and drapes) for the performance of single-shot peripheral regional anesthesia techniques help reduce the incidence of infections related to the technique itself?	10.1 The multidisciplinary expert panel suggests that performing single-shot peripheral regional anesthesia techniques, skin disinfection with 2% chlorhexidine in alcoholic solution, the use of a single-use probe cover, and a no-touch technique are sufficient to reduce the incidence of infections related to the technique itself	
11	Which are the post-procedural monitoring tools for patients undergoing regional anesthesia techniques?	11.1 The multidisciplinary expert panel suggests that patients undergoing subarachnoid and epidural anesthesia should always be clinically reassessed before discharge from the surgical ward. The evaluation should include oxygen saturation, blood pressure, and heart rate. Discharge should only occur after a regression of the sensory block of at least two dermatomes and, in any case, below the T12 dermatome. If intrathecal or epidural opioid is deemed appropriate, it is recommended that the patient’s vital parameters be monitored for at least 30 min before discharge from the surgical ward 11.2 The multidisciplinary expert panel suggests that patient monitoring should not be limited to the surgical ward (operating room and recovery room) but should be a continuous process within the hospital ward, as even severe complications such as neuraxial hematomas can manifest lately. Therefore, it is important to ensure careful neurological surveillance, closely monitoring patients with prolonged sensory and/or motor blocks beyond the expected duration or a recurrence of sensory and/or motor blocks after initial regression	

### Consensus on recommendations

Between April 30, 2024, and May 20, 2024, the expert panel expressed their agreement with the proposed recommendations and good clinical practice statements using an online form.

All recommendations and statements achieved at least 86% agreement within the IQR range of 7–9.

### External review

A preliminary version of the guideline was sent to two external reviewers to assess the content, particularly the interpretation of evidence supporting the recommendations, and to review the methodological approach.

The goal of the external review was to enhance the guideline’s quality, gather feedback on the preliminary recommendations, and evaluate the applicability and feasibility of the evidence. External reviewers were requested to provide comments and observations using a structured form.

## Statements

The forest plots and risk-of-bias assessment whenever available and discussed in the statements are available for consultation as Supplementary Digital Content 1.

## PICO 1

Is it safe to perform a central neuraxial block in patients on cardioaspirin therapy?

### Recommendation 1

*In adult patients on cardioaspirin therapy undergoing RA with neuraxial block, it is recommended to continue cardioaspirin administration, as it does not increase the incidence of spinal hematomas.*

Strength of recommendation:Low.

#### Rationale

Spinal hematomas represent a potentially serious condition that can profoundly impact a patient’s quality of life. However, several studies investigating patients undergoing spinal or epidural anesthesia for orthopedic procedures [8, 9] found no cases of major hemorrhagic events, such as epidural or subarachnoid hematomas. Even studies investigating patients undergoing steroidal injections revealed no occurrences of spinal hematoma as well [10, 11].

An overall analysis of these studies through a random-effects meta-analysis revealed no difference in the risk of spinal hematoma between the two groups. However, due to the limited number of studies and their high consistency, it was not possible to adequately assess publication bias.

## PICO 2

Does the combined use of ultrasound and nerve stimulation (ENS) in peripheral nerve blocks with a motor component in adult patients increase the efficacy and safety of surgical blocks, reducing neurological complications and nerve damage?

### Recommendation 2

*In adult patients undergoing medical procedures requiring RA, the use of dual guidance compared to ENS-only guidance in peripheral nerve blocks with a motor component is recommended, as it reduces the risk of serious neurological complications and/or nerve damage.*

Strength of recommendation: Moderate.

#### Rationale

Across 11 experimental studies, most found no major neurological complications in patients receiving nerve blocks with either dual guidance or ENS alone. Cataldo et al. [12] and Chan et al. [13] reported no significant adverse events, and such complications were not reported in a study [14] though a limited ultrasound visualization was reported. Dhir et al. [15] and Domingo-Triadó et al. [16] observed only minor, self-resolving issues, and no lasting complications in popliteal, interscalene, and supraclavicular blocks [17–20].

However, observational studies by Orebaugh et al. [21, 22] found higher rates of nerve injuries and LAST episodes in ENS-only groups, with fewer cases in dual-guidance groups. Similarly, Zhang et al. [23] observed a higher incidence of neurological complications in ENS-only groups (12%) compared to dual guidance (2%).

Overall, meta-analysis showed no significant difference in severe complication incidence between guidance methods, although limited study numbers and high consistency restricted the ability to assess publication bias adequately.

## PICO 3

Is the use of ultrasound-guided peripheral block techniques safe in adult patients taking direct oral anticoagulants (DOAC)?

### Recommendation 3

*In adult patients taking DOACs, it is suggested to use ultrasound-guided peripheral nerve blocks, as it is safe and does not increase haemorrhagic complications.*

Strength of recommendation: Low.

#### Rationale

The study by Dayan et al. [24] reports major hemorrhagic events in patients undergoing anticoagulant therapy with DOACs undergoing femoral nerve block (54%) compared to patients receiving conventional analgesics only (47%) (relative risk 0.47). In conclusion, only one study reported major bleeding episodes in individuals undergoing medical procedures while on anticoagulant therapy. The study was observational and included a small number of participants. The risk of bias in this study is generally low.

## PICO 4

Can the use of ultrasound-guided techniques increase safety in nerve block procedures in adult patients?

### Recommendation 4

*In adult patients, the use of ultrasound-guided techniques for the performance of peripheral nerve blocks is suggested, as it ensures the safety of the procedure and reduces the risk of complications compared to nerve blocks without ultrasound guidance.*

Strength of recommendation: Moderate

#### Rationale

Studies investigating complications among patients receiving dual guidance compared to patients receiving only ENS revealed no differences among the groups for the sciatic nerve [12, 25] and femoral nerve [26] blocks.

Wang et al.’s study [27] compared bilateral axillary brachial plexus block with ultrasound guidance and ENS guidance, and no episodes of LAST were observed in either group.

No neurological or cardiovascular complications were reported in patients undergoing medial branch spinal nerve block under ultrasound or fluoroscopic guidance [18, 28]. Moreover, a study by Zhang et al. [29] reported no complications related to LAST in an RCT where one group of patients underwent thoracic paravertebral block and another group received a landmark guided block. Ali et al. [30] also reported no local anesthetic toxicity both in subjects undergoing long thoracic nerve block and those receiving thoracic epidural injection.

Li et al. [31] report no LAST cases following both unilateral, bilateral ultrasound-guided transversus abdominis (TAP) block and in patients receiving landmark local anesthetic infiltration.

In a large registry study (26,753 included patients), Bomberg et al. [32] reported only two LAST episodes: one in patients receiving an ultrasound-guided procedure and one in patients receiving an ENS procedure.

However, Zhang et al. [23] reported a lower incidence of LAST in patients undergoing lumbar plexus or sciatic nerve block using dual guidance (2%) compared to the ENS only group (12%).

Additionally, Orebaugh et al. [21] reported five adverse events (seizures) in the group of patients undergoing various nerve blocks with ENS-only guidance and zero in the group undergoing nerve blocks with ultrasound guidance, and in another study by Orebaugh et al. [22], one adverse event (seizure) in the group of patients undergoing ENS-only guidance nerve blocks was reported with no complications in the ultrasound-guided group.

Also, Kaçar et al. [33] do not report episodes of LAST in patients undergoing ultrasound-guided brachial plexus block, while one episode was observed in patients receiving the same block but ENS guided.

In Melnyk et al.’s study [34], only one episode of LAST was described in patients undergoing various ultrasound-guided peripheral nerve blocks, while no episodes occurred in patients receiving the same blocks ENS guided.

Finally, Zhang et al. [35] reported no systemic toxicity in patients undergoing ultrasound-guided peripheral nerve blocks at the extremities, compared to six episodes in patients who underwent the same types of blocks without ultrasound guidance.

When considering the overall difference in risk for developing LAST, a random-effects meta-analysis using the Mantel–Haenszel method does not describe significant difference in the incidence between the two groups, whether they are analyzed individually or combined.

Adequate assessment of publication bias cannot be made due to the limited number of available evidence.

In conclusion, the evidence found regarding adverse events in the form of LAST in patients undergoing medical procedures with or without ultrasound guidance is limited, with a moderate degree of heterogeneity among the comparators examined in the different studies but consistent outcomes. The overall risk of bias in the reviewed evidence is low.

## PICO 5

Can a sterile surgical field (using disinfectants, covers, gloves, and sterile drapes) setup for the execution of neuraxial techniques help reduce the occurrence of infections related to the technique itself?

### Recommendation 5

*In adult patients undergoing epidural anesthesia, the preparation of a sterile surgical field (using antiseptic, covers, gloves, drapes, and mask) is suggested to ensure patient safety.*

Strength of recommendation: Low.

#### Rationale

Birnbach et al. [36] investigated the occurrence of infections in patients undergoing epidural anesthesia, where the skin on the back was cleaned with two different types of disinfectants: polyacrylate iodine and iodopovidone. The positivity rate of these cultures dropped from 90% before disinfection to 3.3% with polyacrylate iodine and to 30% with iodopovidone. After catheter removal (tip of the catheter analysis), the positivity rate in the polyacrylate iodine group was 50%, while in the iodopovidone group it was 96.6%.

Another study by Kasuda et al. [37] also assessed skin cleaning through microbiological cultures in patients undergoing epidural anesthesia. In the iodopovidone group, the cultures’ positivity rate was 25% at the insertion site and 11% at the tip of the catheter. In the chlorhexidine gluconate and ethyl alcohol group, the positivity rate from the insertion site was 24% and 9% at the tip of the catheter.

Kerwat et al. [38] analyzed the incidence of infections through bacterial culture positivity obtained from the catheter tip after disinfection with chlorhexidine gluconate. The positivity rate was 0%, while cultures from the skin at the insertion site showed a positivity rate of 8.6%.

Similarly, Robins et al. [39] examined the incidence of infection in the form of bacterial culture positivity highlighting a higher rate in positivity before the application of a 0.5% chlorhexidine alcohol-based spray (88.5%) compared to the post-disinfection rate (3%).

In conclusion, the evidence regarding adverse events in the form of infections in patients undergoing epidural anesthesia with prior skin disinfection is poor, with consistent outcomes reported across the studies.

## PICO 6

Can a sterile surgical field setup (using disinfectants, covers, gloves, and sterile drapes) for the execution of continuous peripheral regional anesthesia techniques help reduce the development of infections related to the procedure itself?

### Recommendation 6

*In adult patients undergoing continuous peripheral regional anesthesia techniques, the preparation of a sterile surgical field (using disinfectant, covers, gloves, drapes, and mask) for the procedure is suggested to reduce the occurrence of infections and ensure patient and procedural safety.*

Strength of recommendation: Low

#### Rationale

Harkouk et al. [40] investigated the occurrence of infections in patients undergoing peripheral anesthesia, where the skin near the needle insertion site was cleansed with two different types of antiseptics: an alcohol solution with 2% chlorhexidine gluconate and an alcohol solution with 5% iodopovidone. Sterility was assessed through microbiological cultures. The catheter colonization positivity rate was 15.5% after 2% chlorhexidine gluconate disinfection and 32.7% after iodopovidone.

Also, Kerwat et al. [38] investigate the incidence of infections, analyzing bacterial cultures obtained from samples taken from the catheter tip following disinfection with chlorhexidine gluconate. The positivity rate was 0%, while colonization of the epidural insertion site, based on cultures from the skin at the insertion site, showed a positivity rate of 8.6%.

The high heterogeneity of the interventions considered made it unfeasible to conduct the meta-analysis and the subsequent assessment of publication bias.

### Good practice statements

Some recommendations from the present guidelines lack high-quality evidence. For this reason, it will be of paramount importance in the next future to further investigate these topics. In particular, high-quality studies are needed to clarify the safety of RA techniques in anticoagulated patients, the impact of dual guidance in different settings, and the long-term effectiveness of infection prevention strategies. Prospective registries and randomized controlled trials in these areas will enhance future updates of these recommendations.

## PICO 7

Is the use of antiplatelet drugs safe for patients undergoing a dual guidance peripheral nerve block?

*7.1 Superficial peripheral blocks are considered safe in patients on antiplatelet therapy regardless of the dosage or the drug taken.*

*7.2 For deep peripheral nerve blocks in patients undergoing antiplatelet therapy, the same recommendations as neuraxial procedures should be followed regarding the suspension timing of antiplatelet drugs.*

Antiplatelet drugs inhibit platelet aggregation and adhesion, inherently increasing the potential difficulty in bleeding control in case of hemorrhage and hematoma formation. However, in clinical practice, this possibility is rare, and observational studies show the incidence of bleeding complications in patients on antiplatelet therapy undergoing peripheral nerve blocks is uncommon [41] [42].

It is difficult to determine which peripheral blocks are associated with a higher or lower risk, given that studies based on large prospective registries for each specific block are currently unavailable [43].

Nevertheless, it is reasonable, in agreement with other international guidelines, to classify the risk of peripheral blocks according to their anatomical location, thus identifying as high-risk blocks those performed in noncompressible sites [44].

Superficial blocks, by definition, are anesthetic procedures performed in shallow anatomical regions which are easily compressible and amenable to surgical evacuation.

Deep peripheral blocks are performed in hardly compressible sites and where signs of hematoma formation are delayed.

For this reason, and in the absence of specific literature on the subject, it is considered safe to perform superficial peripheral blocks in patients on antiplatelet therapy, provided there is close clinical monitoring for detection of early signs of hematoma formation.

For deep blocks, which may be associated with hematomas that are difficult to diagnose and manage through compression, it seems reasonable to apply the same precautions recommended for neuraxial blocks.

## PICO 8

Is it safe to perform peripheral nerve block techniques with dual guidance in adult patients on anticoagulant therapy?

*8.1 The expert panel suggests that, whenever possible, an appropriate suspension period for anticoagulant medications is always preferable for both deep and superficial peripheral nerve blocks.*

### Rationale

Both peripheral and deep nerve blocks can be associated with hematoma formation, although with different risk profiles. While deep blocks are more frequently associated with neurological damage and life-threatening hemorrhages, superficial blocks are typically characterized by a low risk, with the formation of small, clinically insignificant hematomas [45]. However, in patients undergoing anticoagulant therapy, anecdotal but severe complications have been reported with superficial nerve blocks (hemothorax after supraclavicular block, large chest hematoma after intercostal block [46], and retroperitoneal hematoma after quadratus lumborum block [47]).

Given the limited evidence regarding hemorrhagic and neurological complications, it seems preferable, when possible, to allow an adequate period of suspension for both peripheral and deep nerve blocks.

*8.2 The expert panel suggests that deep peripheral nerve blocks (such as lumbar plexus block) are high-risk procedures for bleeding, and, therefore, in the absence of an adequate suspension period from anticoagulant medications, these blocks cannot be performed safely.*

Deep peripheral nerve blocks may be associated with more severe neurological and hemodynamic complications, mainly because of the non-compressibility of the site, the absence of a superficial “sentinel” hematoma that would allow for rapid identification of bleeding issues, and the proximity to large-calibre arterial and venous vessels as well as the spinal cord [41]. For this reason, the risk–benefit profile appears unacceptable.

*8.3 The expert panel suggests that superficial peripheral nerve blocks can be performed safely in adult patients receiving anticoagulants, even if these medications cannot be suspended.*

Hemorrhagic complications in patients undergoing anticoagulant therapy who are subjected to peripheral nerve blocks are anecdotal [41, 46, 47]. Superficial nerve blocks are usually performed in anatomically compressible regions where a superficial sentinel hematoma can be early detected. Therefore, it is considered feasible to perform superficial peripheral nerve blocks in patients taking anticoagulant medications.

*8.4 The expert panel suggests that the use of dual guidance, compared to not using it, should not influence the anticoagulant suspension time, as there is no evidence that the use of dual guidance is able to reduce haemorrhagic complications in anticoagulated patients.*

Although the rationale is strong and promising, there is currently no study correlating the use of ultrasound with a reduction in neurological or hemorrhagic complications in patients undergoing superficial or deep peripheral nerve blocks. For this reason, there is no evidence to modify the aforementioned recommendations based on the use of ultrasound guidance.

*8.5 The expert panel suggests that all recommendations, precautions, and prescriptions outlined in international guidelines applied to nonurgent cases should be followed also in urgent and emergency situations.*

## PICO 9

Can the use of infusion pressure monitoring techniques, during the performance of peripheral blocks, reduce the onset of neurological complications in adult patients undergoing RA techniques?

*9.1 The multidisciplinary expert panel believes that performing peripheral blocks with infusion pressure monitoring techniques does not reduce occurrence of neurological complications in adult patients undergoing RA techniques.*

The introduction of new technologies for performing RA techniques has increased success rates and reduced neurological complications, improving the quality standards, safety, and patient outcomes. From nerve research guided by anatomical landmarks and the evocation of paraesthesias, the technique has evolved to neurostimulation and, more recently, the use of ultrasonography.

Performing peripheral blocks with both ultrasound guidance and neurostimulation requires proper training, anatomical knowledge, and manual skills.

The correct execution of a nerve block requires extreme movement precision, as even a millimeter of error in the conduction and positioning of the needle tip can reduce the success rates of the block and lead to complications.

The incidence of intraneural injection after ENS or the occurrence of paraesthesias is not well documented. Ultrasound guidance for peripheral nerve blocks has enhanced understanding of the needle-nerve interaction during ENS or local anesthetic injection.

Robards et al. [48] demonstrated that intraneural needle placement and intraneural injection of local anesthetic during popliteal sciatic nerve block are not rare events, even when using low-current ENS techniques.

Several clinical studies have suggested that a motor response may be absent even when the needle is so close to a nerve it causes paraesthesias [49–52], meaning that that when only a ENS is used to locate the sciatic nerve in the popliteal fossa, the motor response may not be elicited even with direct needle-nerve contact, necessitating further attempts to trigger the motor response.

Varobieff et al. [53] showed that pressure monitoring may have good sensitivity but not specificity, whereas ENS appears to be specific but not sensitive. The combination of electrical stimulation and pressure monitoring could provide valuable information about the needle tip’s position in contact with the nerve or even its intraneural placement.

However, recent studies have shown that fascicles have additional protection, as do the nerves. Therefore, compared to the traditional model, the nerve should be considered as the sum of smaller nerves (individual fascicles) protected by collagen.

## PICO 10

Can the preparation of a sterile surgical field (disinfectant, probe covers, gloves, and drapes) for the performance of single-shot peripheral regional anesthesia techniques help reduce the incidence of infections related to the technique itself?

*10.1 The multidisciplinary expert panel suggests that performing single-shot peripheral regional anesthesia techniques, skin disinfection with 2% chlorhexidine in alcoholic solution, the use of a single-use probe cover, and a no-touch technique are sufficient to reduce the incidence of infections related to the technique itself.*

Multiple variables could play a role in the development of an infection, depending on both intrinsic patient factors (such as diabetes or immunosuppression) and extrinsic factors related to the procedure. Aseptic rules are usually defined by institutional protocols, but several aspects remain variable, such as technique, the use of sterile or single-use materials, sterile preparation of the injected drugs, and appropriate antibiotic prophylaxis timing [54, 55]. Many of the guidelines and recommendations proposed or described come from surgical literature and would require proper extrapolation when applied to RA.

Infectious complications related to single-shot peripheral blocks are extremely rare [56], and no cases describing infections associated with a single ultrasound-guided peripheral nerve block have been reported in literature.

Adequate hand hygiene, according to the five moments defined by the WHO, is a key component in ensuring sterile execution of anesthesiological procedures [57].

Typically, ultrasound-guided techniques involve a single-needle insertion into the skin, comparable to an intramuscular injection, and are less invasive than peripheral venous catheter placement. For these procedures, sterility protocols require adequate skin disinfection with 0.5% chlorhexidine solution and the use of sterile needles and materials [58]. A single chlorhexidine application is sufficient to eliminate skin microorganisms [59]. In a study that considered 1134 tunnelled catheters in the interscalene site, it was observed that extending the duration of skin disinfection time with 70% ethanol solution to 10 min significantly reduced the incidence of infections [60].

The British guidelines from the Association of Anaesthetists recommend allowing the ethanol solution to dry before palpating or puncturing the skin [61]. In many clinical settings, disinfection is now performed using a spray formulation. The “no-touch” technique ensures no direct contact between the operator and the patient’s skin or the penetrating part of the needle [62]. Australian anesthesiologists’ guidelines for single-shot non-neuraxial blocks require skin disinfection, clean hands and gloves, single-use needles and syringes, and the no-touch technique [63]. It is also considered good practice to avoid passing through the gel applied between the probe and the skin with the needle [64].

According to the Spaulding classification of medical devices, the ultrasound machine used for single-shot peripheral LRA should be classified as a noncritical device (instruments and objects that only come into contact with intact skin) and only require basic cleaning based on manufacturer recommendations [65]. A decade-long hospital experience in Toronto, involving over 7476 single-shot ultrasound-guided procedures, confirmed an extremely low infection rate when cleaning the equipment with surface disinfectant wipes, allowing it to air dry, and using a sterile probe cover [66]. To avoid contamination or cross-infections between patients, the ultrasound probe should always be covered with a single-use probe cover, and the cable should be managed to maintain the sterility of the procedural area [67].

With the exception of antiseptic solutions, the various aseptic components for single peripheral blocks have been poorly investigated [68], and never prospectively, so scientific evidence is lacking. Much of the infection prevention guidelines in anesthesia are pulled out from surgical procedures and are primarily recommended for central venous access, continuous perineural catheters, and single or continuous neuraxial blocks. These guidelines are often too rigid, time-consuming, and expensive.

On the other hand, there is ample clinical or practical evidence where, in the absence of well-established procedures, the current widespread clinical practice has proven to be both effective and safe for patients. An additional consideration for adequate sterility management is the amount of waste generated during the execution of a peripheral block and the subsequent environmental pollution. Efforts should be made to avoid producing unnecessary waste. Along with the appropriate use of materials, including packaging, there is also a need to consider the disposal of waste generated, which is not always aligned with the actual requirements for performing a sterile block. Operating rooms account for a quarter of all hospital waste, with up to 25% of which coming from anesthesia. By applying the 6 Rs (Rethink, Refuse, Reduce, Reuse, Recycle, and Research) to reduce the carbon footprint and slow down the global warming rate, a shift in mindset is needed while still keeping the primary focus on doing the best for the patients we take care of [69].

## PICO 11

Which are the post-procedural monitoring tools for patients undergoing regional anesthesia techniques?

*11.1 The multidisciplinary expert panel suggests that patients undergoing subarachnoid and epidural anesthesia should always be clinically reassessed before discharge from the surgical ward. The evaluation should include oxygen saturation, blood pressure, and heart rate. Discharge should only occur after a regression of the sensory block of at least two dermatomes and, in any case, below the T12 dermatome.*

If intrathecal or epidural opioid is deemed appropriate, it is recommended that the patient’s vital parameters be monitored for at least 30 min before discharge from the surgical ward.

*11.2 The multidisciplinary expert panel suggests that patient monitoring should not be limited to the surgical ward (operating room and recovery room) but should be a continuous process within the hospital ward, as even severe complications such as neuraxial hematomas can manifest lately. Therefore, it is important to ensure careful neurological surveillance, closely monitoring patients with prolonged sensory and/or motor blocks beyond the expected duration or a recurrence of sensory and/or motor blocks after initial regression.*

Patients undergoing subarachnoid anesthesia should meet the following criteria before being discharged from the operating room:

- The sensory block should regress by two dermatomes from the level where the block was initially reached and be below the T12 dermatome.
- Motor function should be assessed using the Bromage scale, with a minimum score of 2.

Patients with continuous epidural analgesia should be discharged after 1 h of monitoring if they meet sensory block regression criteria.

Historically, the incidence of spinal epidural hematoma following neuraxial anesthesia has been estimated to be less than 1 in 150,000 epidural placements and less than 1 in 220,000 spinal anesthetics. However, this incidence varies widely depending on the patient population.

Recent studies on the incidence of spinal hematoma risk in patients without obvious risk factors have shown an increase to 1:18,000 after epidural anesthesia and 1:3600, or even 1:1000, in elderly patients undergoing lower limb surgery [70, 71].

It would be advisable to control the motor and sensory function recovery within 2 h from the block and check for eventual associated symptoms such as weakness, numbness, and urinary or fecal incontinence.

The reappearance of sensory or motor deficits hours after subarachnoid or epidural block has regressed (with or without back pain) is highly suggestive of a spinal or epidural hematoma and should be considered and treated as such until proven otherwise.

Neurological recovery may occur if surgery and decompression are performed within 36 h of complete motor deficit and within 48 h of partial deficit.

Additionally, when possible, the use of echogenic needles should be assessed for its potential role in reducing complications. The expert panel believes that in the absence of definitive evidence, it is reasonable to use echogenic needles to allow for better visualization of the needle’s various parts, especially the tip. Recognizing the different parts of the needle would implicitly increase safety during the procedure, thereby reducing complications.

### Economic impact

Economic implications, in terms of costs and the allocation of financial resources required for the implementation of the recommendations outlined in the guidelines, are beyond the scope of this document.

## Supplementary Information

Supplementary Material 1. Digital Content 1: Forest Plots and Risk of bias analysis PICO 1 - Is it safe to perform a central neuraxial block in patients on cardioaspirin therapy?. PICO 2 - Does the combined use of ultrasound and nerve stimulation (ENS) in peripheral nerve blocks with a motor component in adult patients increase the efficacy and safety of surgical blocks, reducing neurological and nerve damage? PICO 3 - Is the use of ultrasound-guided peripheral block techniques safe in adult patients taking Direct Oral Anticoagulants (DOAC)? PICO 4 - Can the use of ultrasound-guided techniques increase safety in nerve block procedures on adult patients? PICO 5- Can a sterile surgical field (using disinfectants, covers, gloves, and sterile drapes) setup for the execution of neuraxial techniques, help reduce the occurrence of infections related to the technique itself? PICO 6 - In adult patients (≥18 years) undergoing continuous peripheral loco-regional techniques, the preparation of a sterile surgical field (using disinfectant, covers, gloves, drapes, and mask) for the procedure is suggested to reduce the occurrence of infections and ensure patient and procedural safety. PICO 7 - Is the use of antiplatelet drugs safe for patients undergoing an ultrasound + ENS guided (dual guidance) peripheral nerve block? PICO 8 - Is it safe to perform peripheral nerve block techniques with ultrasound guidance and nerve stimulation (dual guidance) in adult patients on anticoagulant therapy? PICO 9 - Can the use of infusion pressure monitoring techniques, during the performance of peripheral blocks, reduce the onset of neurological complications in adult patients undergoing regional anesthesia techniques? PICO 10 - Can the preparation of a sterile surgical field (disinfectant, probe covers, gloves, and drapes) for the performance of single-shot peripheral loco-regional techniques help reduce the incidence of infections related to the technique itself? PICO 11 - Which are the post-procedural monitoring tools for patients undergoing loco-regional techniques?

## Data Availability

No datasets were generated or analysed during the current study.
