# Supplementary material for: Enhancing Safety in Regional Anesthesia: Guidelines from the Italian Society of Anesthesia, Analgesia, Resuscitation and Intensive Care (SIAARTI)
Source: J Anesth Analg Crit Care. 2025 May 14;5:26. doi: 10.1186/s44158-025-00245-y (PMC12076962; doi:10.1186/s44158-025-00245-y)
Supplement: Supplementary file 1 — Supplementary Material 1. Digital Content 1: Forest Plots and Risk of bias analysis PICO 1 - Is it safe to perform a central neuraxial block in patients on cardioaspirin therapy?. PICO 2 - Does the combined use of ultrasound and nerve stimulation (ENS) in peripheral nerve blocks with a motor component in adult patients increase the efficacy and safety of surgical blocks, reducing neurological and nerve damage? PICO 3 - Is the use of ultrasound-guided peripheral block techniques safe in adult patients taking Direct Oral Anticoagulants (DOAC)? PICO 4 - Can the use of ultrasound-guided techniques increase safety in nerve block procedures on adult patients? PICO 5- Can a sterile surgical field (using disinfectants, covers, gloves, and sterile drapes) setup for the execution of neuraxial techniques, help reduce the occurrence of infections related to the technique itself? PICO 6 - In adult patients (≥18 years) undergoing continuous peripheral loco-regional techniques, the preparation of a sterile surgical field (using disinfectant, covers, gloves, drapes, and mask) for the procedure is suggested to reduce the occurrence of infections and ensure patient and procedural safety. PICO 7 - Is the use of antiplatelet drugs safe for patients undergoing an ultrasound + ENS guided (dual guidance) peripheral nerve block? PICO 8 - Is it safe to perform peripheral nerve block techniques with ultrasound guidance and nerve stimulation (dual guidance) in adult patients on anticoagulant therapy? PICO 9 - Can the use of infusion pressure monitoring techniques, during the performance of peripheral blocks, reduce the onset of neurological complications in adult patients undergoing regional anesthesia techniques? PICO 10 - Can the preparation of a sterile surgical field (disinfectant, probe covers, gloves, and drapes) for the performance of single-shot peripheral loco-regional techniques help reduce the incidence of infections related to the technique itself? PICO 11 - Which are the post-pro [file 44158_2025_245_MOESM1_ESM.pdf]

Supplementary Digital Content 1 : Forest Plots and Risk of bias analysis

PICO 1 - *Is it safe to perform a central neuraxial block in patients on cardioaspirin therapy?*

Forest plot PICO 1

	local anesthesia in aspirin treatment		local anesthesia in other antiplatelet treatment			Risk Difference
Study or Subgroup	Events	Total	Events	Total	Weight	M-H, Random, 95% CI
Horlocker 1990	0	209	0	232	33.8%	0.00 [-0.01, 0.01]
Horlocker 1995	0	193	0	225	30.1%	0.00 [-0.01, 0.01]
Horlocker 2002	0	134	0	250	19.7%	0.00 [-0.01, 0.01]
Park 2017	0	108	0	1534	16.5%	0.00 [-0.01, 0.01]
**Total (95% CI)**		**644**		**2241**	**100.0%**	**0.00 [-0.01, 0.01]**
Total events	0		0			

Heterogeneity: $Tau^2 = 0.00$; $Chi^2 = 0.00$, df = 3 (P = 1.00); $I^2 = 0\%$
Test for overall effect: Z = 0.00 (P = 1.00)

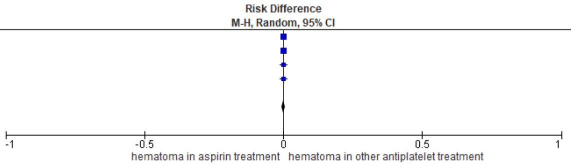

Risk of bias – PICO 1

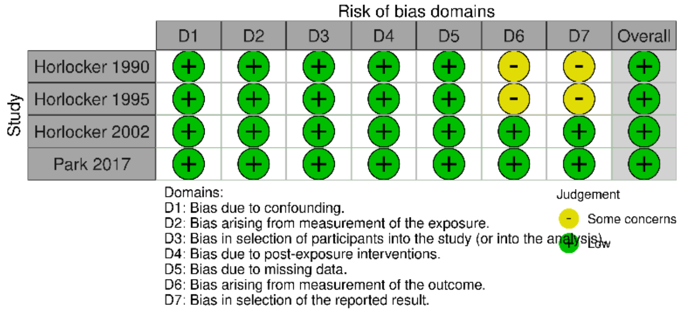

PICO 2 - ***Does the combined use of ultrasound and nerve stimulation (ENS) in peripheral nerve blocks with a motor component in adult patients increase the efficacy and safety of surgical blocks, reducing neurological and nerve damage?***

Forest plot PICO 2

Study or Subgroup	dual guidance Events	dual guidance Total	ENS only Events	ENS only Total	Weight	Risk Difference M-H, Random, 95% CI
**1.2.1 RCTs**						
Amin 2015	0	18	0	18	0.0%	0.00 [-0.10, 0.10]
Cataldo 2012	0	35	0	35	0.1%	0.00 [-0.05, 0.05]
Chan 2007	0	62	0	62	0.2%	0.00 [-0.03, 0.03]
Dhir 2008	0	22	0	18	0.0%	0.00 [-0.09, 0.09]
Domingo-Triadó 2007	0	30	0	31	0.0%	0.00 [-0.06, 0.06]
Dufour 2008	0	26	0	25	0.0%	0.00 [-0.07, 0.07]
Kolny 2017	0	100	0	96	0.5%	0.00 [-0.02, 0.02]
Salem 2012	0	30	0	30	0.0%	0.00 [-0.06, 0.06]
Williams 2003	0	100	0	109	0.5%	0.00 [-0.02, 0.02]
Xiao 2020	0	22	0	22	0.0%	0.00 [-0.08, 0.08]
Zhang 2019	0	100	0	100	0.5%	0.00 [-0.02, 0.02]
**Subtotal (95% CI)**		**545**		**546**	**1.9%**	**0.00 [-0.01, 0.01]**
Total events	0		0			
Heterogeneity: Tau² = 0.00; Chi² = 0.00, df = 10 (P = 1.00); I² = 0%						
Test for overall effect: Z = 0.00 (P = 1.00)						
**1.2.2 Observational studies**						
Orebaugh 2009	0	2146	8	3290	52.9%	-0.00 [-0.00, -0.00]
Orebaugh 2012	1	7092	5	2209	45.2%	-0.00 [-0.00, -0.00]
**Subtotal (95% CI)**		**9238**		**5499**	**98.1%**	**-0.00 [-0.00, -0.00]**
Total events	1		13			
Heterogeneity: Tau² = 0.00; Chi² = 0.05, df = 1 (P = 0.82); I² = 0%						
Test for overall effect: Z = 3.30 (P = 0.0010)						
**Total (95% CI)**		**9783**		**6045**	**100.0%**	**-0.00 [-0.00, -0.00]**
Total events	1		13			
Heterogeneity: Tau² = 0.00; Chi² = 0.32, df = 12 (P = 1.00); I² = 0%						
Test for overall effect: Z = 3.27 (P = 0.001)						
Test for subgroup differences: Chi² = 0.21, df = 1 (P = 0.65), I² = 0%						

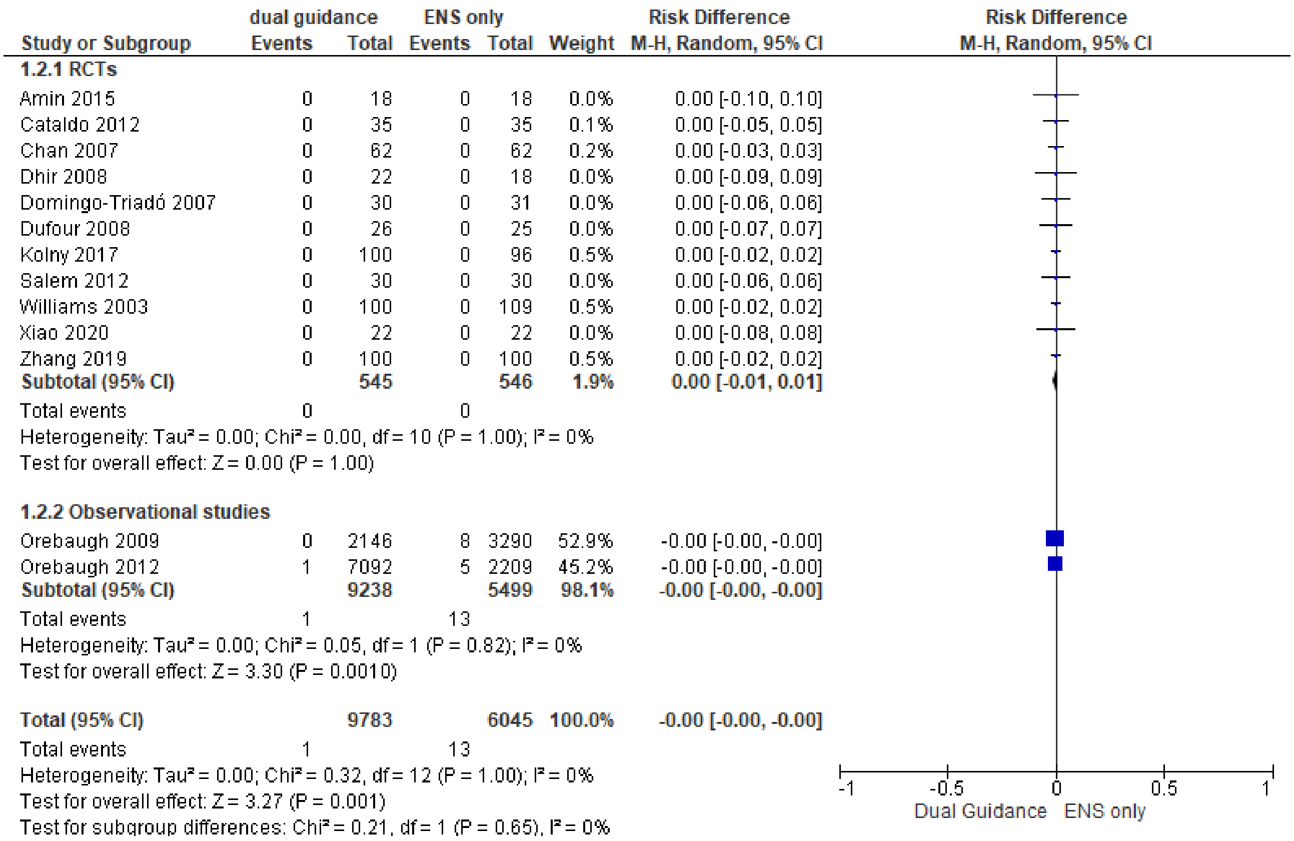

Risk of bias – PICO 2

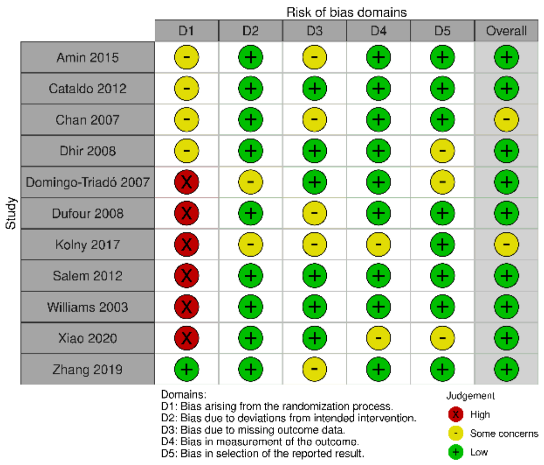

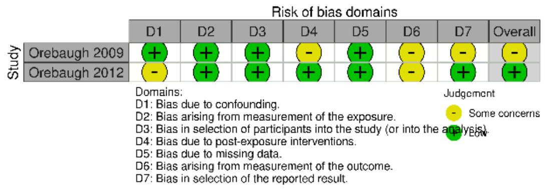

PICO 3 - *Is the use of ultrasound-guided peripheral block techniques safe in adult patients taking Direct Oral Anticoagulants (DOAC) ?*

Risk of bias – PICO 3

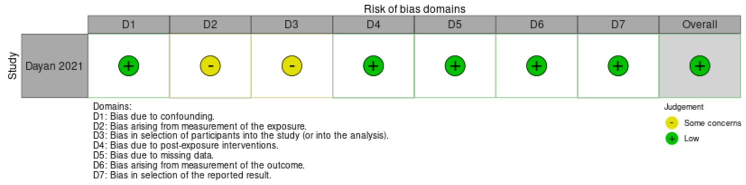

PICO 4 - *Can the use of ultrasound-guided techniques increase safety in nerve block procedures on adult patients?*

Forest plot PICO 4

Study or Subgroup	Ultrasound-guided block(s) Events	Total	Not ultrasound-guided block(s) Events	Total	Weight	Risk Difference M-H, Random, 95% CI
**1.1.1 RCTs**						
Ali 2021	0	20	0	20	0.0%	0.00 [-0.09, 0.09]
Cataldo 2012	0	35	0	35	0.0%	0.00 [-0.05, 0.05]
Li 2018	0	47	0	22	0.0%	0.00 [-0.07, 0.07]
Mouzi 2016	0	8	0	8	0.0%	0.00 [-0.21, 0.21]
Zhang 2019	12	100	4	100	0.0%	0.08 [0.01, 0.15]
Zhang 2020	0	30	0	26	0.0%	0.00 [-0.07, 0.07]
**Subtotal (95% CI)**		**240**		**211**	**0.0%**	**0.01 [-0.02, 0.05]**
Total events	12		4			
Heterogeneity: Tau² = 0.00; Chi² = 6.02, df = 5 (P = 0.30); I² = 17%						
Test for overall effect: Z = 0.79 (P = 0.43)						
**1.1.2 Observational studies**						
Barrington 2013	12	20401	10	4745	8.3%	-0.00 [-0.00, -0.00]
Bomberg 2018	0	10380	0	8180	33.5%	0.00 [-0.00, 0.00]
Han 2017	0	68	0	78	0.0%	0.00 [-0.03, 0.03]
Jang 2020	0	44	0	78	0.0%	0.00 [-0.04, 0.04]
Kaçar 2018	0	118	1	68	0.0%	-0.01 [-0.05, 0.02]
Melnyk 2018	0	47	0	22	0.0%	0.00 [-0.07, 0.07]
Orebaugh 2009	0	3290	0	2146	17.3%	0.00 [-0.00, 0.00]
Orebaugh 2012	0	2209	0	7092	20.2%	0.00 [-0.00, 0.00]
Wang 2016	0	60	0	60	0.0%	0.00 [-0.03, 0.03]
Zhang 2021	0	8118	6	8118	20.7%	-0.00 [-0.00, -0.00]
**Subtotal (95% CI)**		**44735**		**30587**	**100.0%**	**-0.00 [-0.00, 0.00]**
Total events	12		17			
Heterogeneity: Tau² = 0.00; Chi² = 38.62, df = 9 (P < 0.0001); I² = 77%						
Test for overall effect: Z = 0.94 (P = 0.34)						
**Total (95% CI)**		**44975**		**30798**	**100.0%**	**-0.00 [-0.00, 0.00]**
Total events	24		21			
Heterogeneity: Tau² = 0.00; Chi² = 21.33, df = 15 (P = 0.13); I² = 30%						
Test for overall effect: Z = 1.23 (P = 0.22)						
Test for subgroup differences: Chi² = 0.66, df = 1 (P = 0.42), I² = 0%						

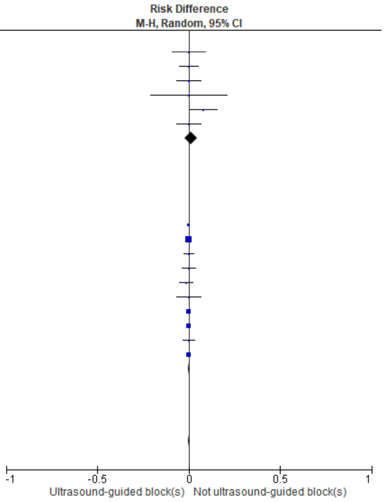

Study or Subgroup	Ultrasound-guided block(s) Events	Total	Fluoroscopy-guided block(s) Events	Total	Weight	Risk Difference M-H, Random, 95% CI
Han 2017	0	68	0	78	63.8%	0.00 [-0.03, 0.03]
Jang 2020	0	44	0	78	36.2%	0.00 [-0.04, 0.04]
**Total (95% CI)**		**112**		**156**	**100.0%**	**0.00 [-0.02, 0.02]**
Total events	0		0			
Heterogeneity: Tau² = 0.00; Chi² = 0.00, df = 1 (P = 1.00); I² = 0%						
Test for overall effect: Z = 0.00 (P = 1.00)						

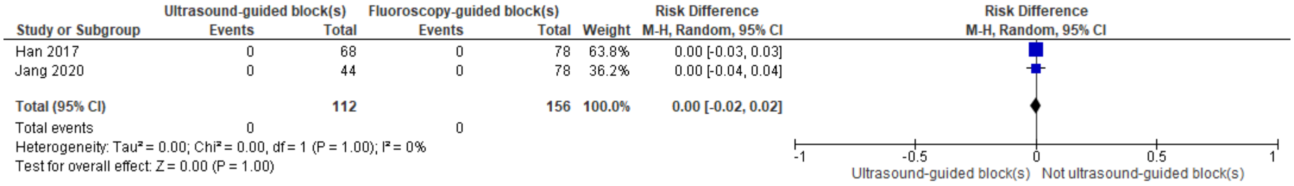

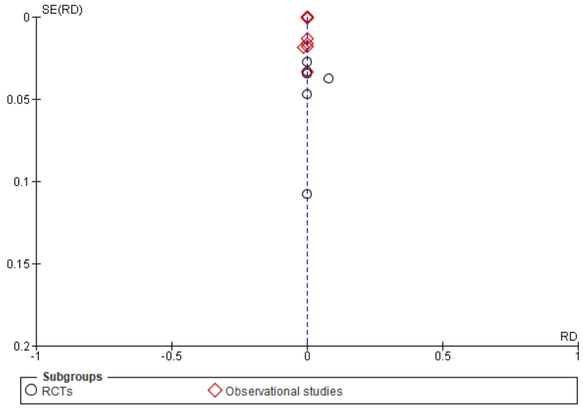

Risk of bias – PICO 4

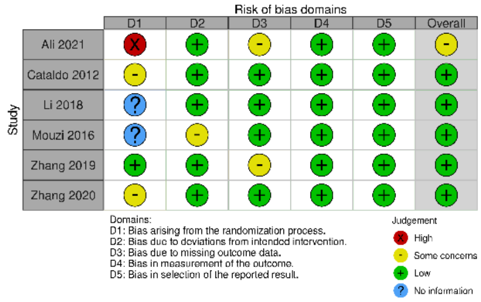

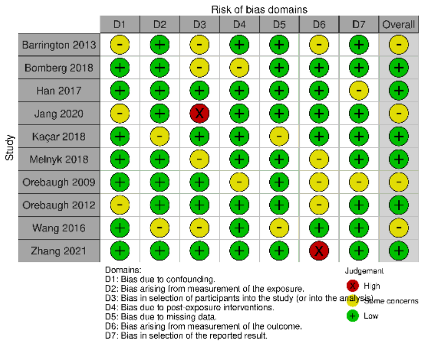

PICO 5- ***Can a sterile surgical field (using disinfectants, covers, gloves, and sterile drapes) setup for the execution of neuraxial techniques, help reduce the occurrence of infections related to the technique itself?***

Risk of bias – PICO 5

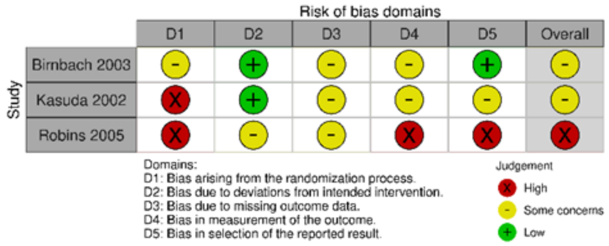

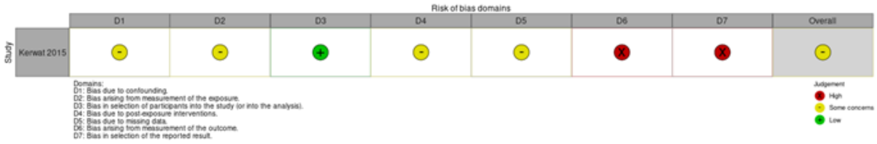

PICO 6 - ***In adult patients (≥18 years) undergoing continuous peripheral loco-regional techniques, the preparation of a sterile surgical field (using disinfectant, covers, gloves, drapes, and mask) for the procedure is suggested to reduce the occurrence of infections and ensure patient and procedural safety.***

Risk of bias – PICO 6

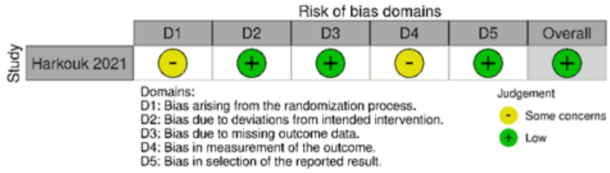

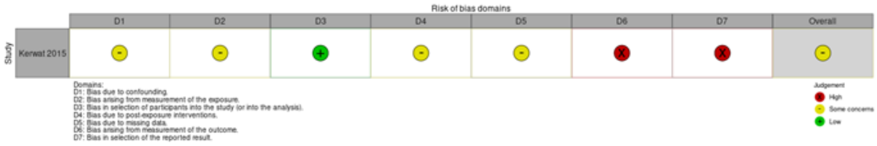

PICO 7 - ***Is the use of antiplatelet drugs safe for patients undergoing an ultrasound + ENS guided (dual guidance) peripheral nerve block?***

PICO 8 - ***Is it safe to perform peripheral nerve block techniques with ultrasound guidance and nerve stimulation (dual guidance) in adult patients on anticoagulant therapy?***

PICO 9 - ***Can the use of infusion pressure monitoring techniques, during the performance of peripheral blocks, reduce the onset of neurological complications in adult patients undergoing regional anesthesia techniques?***

PICO 10 - ***Can the preparation of a sterile surgical field (disinfectant, probe covers, gloves, and drapes) for the performance of single-shot peripheral loco-regional techniques help reduce the incidence of infections related to the technique itself?***

PICO 11 - ***Which are the post-procedural monitoring tools for patients undergoing loco-regional techniques?***
